# Intrinsic Oncogenic Function of Intracellular Connexin26 Protein in Head and Neck Squamous Cell Carcinoma Cells

**DOI:** 10.3390/ijms19072134

**Published:** 2018-07-23

**Authors:** Nobuko Iikawa, Yohei Yamamoto, Yohei Kawasaki, Aki Nishijima-Matsunobu, Maya Suzuki, Takechiyo Yamada, Yasufumi Omori

**Affiliations:** 1 Department of Molecular and Tumour Pathology, Akita University Graduate School of Medicine, Akita 010-8543, Japan; iikawan@med.akita-u.ac.jp (N.I.); cificap@med.akita-u.ac.jp (Y.Y.); akinishijima@med.akita-u.ac.jp (A.N.-M.); maya@med.akita-u.ac.jp (M.S.); 2 Department of Otorhinolaryngology and Head-and-Neck Surgery, Akita University Graduate School of Medicine, Akita 010-8543, Japan; kawa0807@med.akita-u.ac.jp (Y.K.); ymdtkcy@med.akita-u.ac.jp (T.Y.)

**Keywords:** gap junction, cell-cell communication, connexin26, head and neck squamous cell carcinoma, ER-Golgi retention signal, cancer progression

## Abstract

It has long been known that the gap junction is down-regulated in many tumours. One of the downregulation mechanisms is the translocation of connexin, a gap junction protein, from cell membrane into cytoplasm, nucleus, or Golgi apparatus. Interestingly, as tumours progress and reinforce their malignant phenotype, the amount of aberrantly-localised connexin increases in different malignant tumours including oesophageal squamous cell carcinoma, thus suggesting that such an aberrantly-localised connexin should be oncogenic, although gap junctional connexins are often tumour-suppressive. To define the dual roles of connexin in head and neck squamous cell carcinoma (HNSCC), we introduced the wild-type connexin26 (wtCx26) or the mutant Cx26 (icCx26) gene, the product of which carries the amino acid sequence AKKFF, an endoplasmic reticulum-Golgi retention signal, at the C-terminus and is not sorted to cell membrane, into the human FaDu hypopharyngeal cancer cell line that had severely impaired the expression of connexin during carcinogenesis. wtCx26 protein was trafficked to the cell membrane and formed gap junction, which successfully exerted cell-cell communication. On the other hand, the icCx26 protein was co-localised with a Golgi marker, as revealed by immunofluorescence, and thus was retained on the way to the cell membrane. While the forced expression of wtCx26 suppressed both cell proliferation in vitro and tumorigenicity in mice in vivo, icCx26 significantly enhanced both cell proliferation and tumorigenicity compared with the mock control clones, indicating that an excessive accumulation of connexin protein in intracellular domains should be involved in cancer progression and that restoration of proper subcellular sorting of connexin might be a therapeutic strategy to control HNSCC.

## 1. Introduction

Gap junction (GJ) is an intercellular channel directly connecting the cytoplasms of two adjacent cells, which then exchange water-soluble small molecules (<1 kDa) through GJ [1]. Serving as a tool for cell-cell communication, GJ plays essential roles in the homeostasis of cellular society. To the contrary, it has been well known that failure in gap junctional intercellular communication (GJIC) is profoundly involved in serious disorders and diseases of various organs, including arrhythmia [2], sensorineural hearing loss [3], Charcot-Marie-Tooth disease [4], and even cancers [5]. A gap junctional channel comprises two membrane-integrated hemichannels provided by each of two adjacent cells, and each hemichannel is composed of hexameric connexin molecules to become functional. In mammals, more than 20 molecular species have so far been identified in a connexin protein family [6,7]. Usually, several different connexin proteins are co-expressed in a single cell and the combination of the expressed connexin proteins varies tissue to tissue, i.e., while the keratinocytes of the epidermis express many connexin proteins such as connexin26, 30, 30.3, 31.1, 32, 43, and 45 [8], the cardiomyocytes express connexin40, 43, and 45 [9].

A large number of reports from us and others have established that GJIC suppresses tumour promotion in carcinogenesis by restraining the cancer-initiated cells in cellular society and that downregulation of GJIC leads to cancer development, as proven by a considerable number of experiments both in vitro and in vivo [10]. In this context, since the connexin protein localised in cytoplasm cannot participate in GJ formation, translocation of connexin protein from cell membrane to cytoplasm is considered to be loss-of-function of GJ in terms of tumour suppression. However, there is a growing body of evidence indicating that an excessive accumulation of connexin protein in cytoplasm and/or organelles enhances cancer progression such as invasion and metastasis [11,12,13,14]. Notably in squamous cell carcinoma of the head and neck and the oesophagus, the expression level of connexin26 (Cx26) in various intracellular domains correlates to the grade of malignancy or the extent of lymph node metastasis [15,16]. Furthermore, we have previously reported that accumulation of connexin32 (Cx32) in Golgi apparatus increases cancer stem cells in number and enhances the metastatic ability of the cell lines derived from human hepatocellular carcinoma [17].

In the present study, to define the roles of connexin protein localised in a Golgi area in the malignant phenotype of human head and neck squamous cell carcinoma (HNSCC), we transduced human FaDu hypopharyngeal cancer cells with the retrovirus vector carrying the mutant Cx26 cDNA which encoded intracellular Cx26 (icCx26) protein and compared their cancerous behaviours with those of the clones transduced with the wild-type Cx26 (wtCx26) or the empty vector. Our different analyses finally indicated that while wtCx26 protein integrated into GJ at cell membrane functioned tumour-suppressively, icCx26 protein rather reinforced malignant phenotype in FaDu cells.

## 2. Results

### 2.1. AKKFF Amino Acid Sequence Successfully Retains Cx26 Protein in a Golgi Area in FaDu Cells

**Figure 1:**
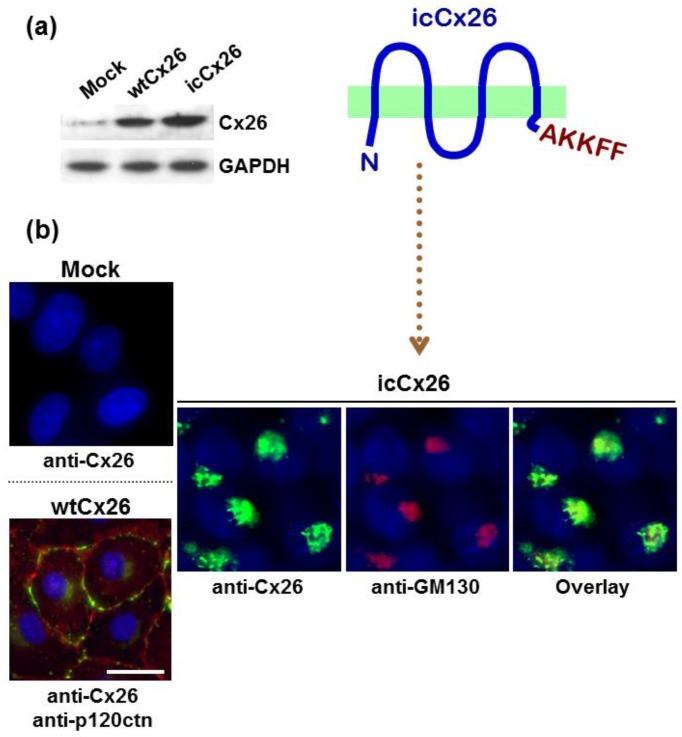
Expression and subcellular localisation of wtCx26 or icCx26 protein in the FaDu clones retrovirally-transduced by each construct examined. (**a**) Immunoblotting of Cx26 protein expressed in the wtCx26, icCx26, and mock clones. The expression of glyceraldehyde-3-phosphate dehydrogenase (GAPDH) was examined as a loading control. (**b**) Indirect immunofluorescence of Cx26, p120catenin (p120ctn), and GM130 proteins in the FaDu clones. The fluorescent signals of p120ctn protein were visualised by Alexa-568 (orange to red) and indicate a juxtamembrane area in the wtCx26 clone. Nuclei were stained with diamidine phenylindole dihydrochloride (DAPI). Note that signals of both Cx26 and GM130 proteins are co-localised in the icCx26 clone (overlay). Scale bar, 20 µm.

**Figure A1:**
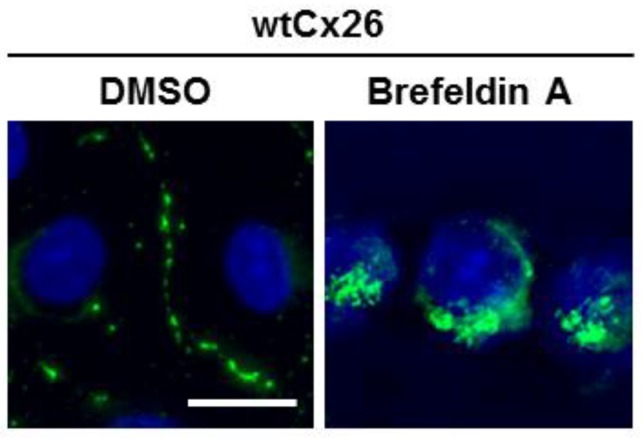
Effect of Brefeldin A on wtCx26-mdiated GJ plaques. Indirect immunofluorescence of Cx26 protein in wtCx26 clone in the presence or absence of 5 µg/mL Brefeldin A/DMSO. The fluorescent signals of Cx26 protein were visualised by Alexa-488. Nuclei were stained with DAPI. Scale bar, 20 µm.

As previously reported, an excessive accumulation of Cx32 protein in Golgi apparatus reinforces the malignant phenotype of human hepatoma HuH7 cells, suggesting that connexin protein in Golgi apparatus might have a distinct function from GJ [18]. Thus, to define the roles of intracellular connexin protein in the malignant phenotype in the context of HNSCC, the mutant Cx26 protein in which the amino acid sequence AKKFF, an endoplasmic reticulum (ER)-Golgi retention signal [19], was added to the C-terminus was overexpressed in human FaDu hypopharyngeal HNSCC cells by retroviral transduction. As shown in Figure 1a, the control mock-transduced FaDu cells express a negligible amount of endogenous Cx26 protein. On the other hand, the clones transduced by either wtCx26 or icCx26 construct overexpress respective corresponding proteins. Consistently with the immunoblotting, wtCx26 protein successfully provides punctuate strong fluorescent signals at a cell-cell contact area, indicating an efficient formation of GJ plaques (Figure 1b). In contrast, the icCx26 protein covalently-conjugated with the ER-Golgi retention signal fails to locate in cell membrane and is co-localised with a Golgi marker GM130, indicating a dense accumulation of icCx26 protein in a Golgi area. The amino acid sequence AKKFF we used as an ER-Golgi retention signal has been reported to target connexin43 (Cx43) protein to the ER-Golgi intermediate compartment (ERGIC) [19]. As shown in Figure 1b, immunofluorescent signals given by icCx26 protein appear much larger than ERGIC [20]. We thus interpret that icCx26 protein is localised not only in ERGIC but also inside or on Golgi apparatus. It has been known that Cx26, unlike Cx32 and Cx43, takes an alternative pathway instead of the secretory pathway as a membrane trafficking route [21]. However, as shown in Figure A1, GJ plaques composed of wtCx26 are disrupted in the presence of Brefeldin A, suggesting that wtCx26 could take the secretory pathway in our FaDu cells as observed in mouse keratinocytes [22].

### 2.2. wtCx26 but Not icCx26 Protein Has the Ability to Exert GJIC

**Figure 2:**
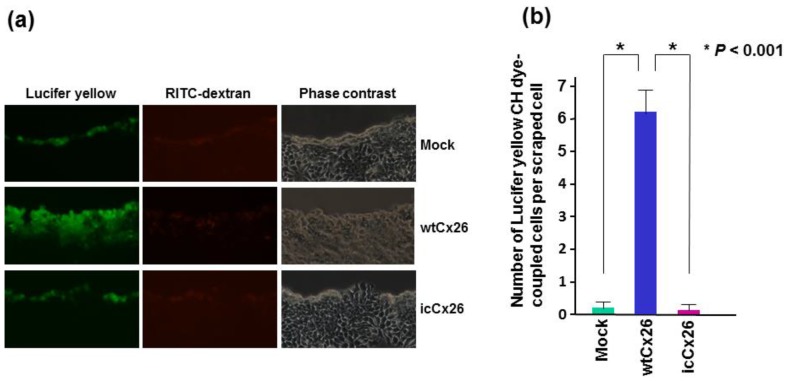
Scrape-loading dye-transfer assay to measure GJIC ability. The wtCx26, icCx26, and mock clones were soaked in a cocktail of Lucifer yellow CH and RITC-dextran, scraped by a micropipette tip, and observed under a fluorescence microscope after 5 min of incubation. (**a**) Representative micrographs of 3 different clones. The same fields of each clone were captured. Note that dye-coupled cells with Lucifer yellow CH were observed only in the wtCx26 clone. (**b**) Histogram showing the mean GJIC capacity of each clone. Error bars represent the SD (*n* = 6).

As mentioned above, wtCx26 protein is sorted to cell membrane and is capable of forming GJ plaques in a cell-cell contact area of FaDu cells. To examine whether the wild type-mediated GJs are indeed functional in FaDu cells, we performed a scrape loading dye-coupling assay. As shown in Figure 2a, while the primarily-scraped cells are co-stained by rhodamine B isothiocyanate (RITC)-dextran and Lucifer yellow in all the clones examined, the clone transduced with wtCx26 but neither icCx26 nor the mock construct is positive only for Lucifer yellow in a zone adjacent to RITC-positive cells. Since, unlike RITC-dextran, Lucifer yellow can pass through GJ, the cells in the Lucifer yellow-positive zone are considered to have received the dye through functional GJs. As predicted, these results clearly indicate that icCx26 protein cannot contribute to GJ formation in cell membrane and is thus non-functional as a GJ protein (Figure 2b).

### 2.3. wtCx26 and icCx26 Proteins Regulate Cell Proliferation and Invasion in a Reciprocal Manner

**Figure 3:**
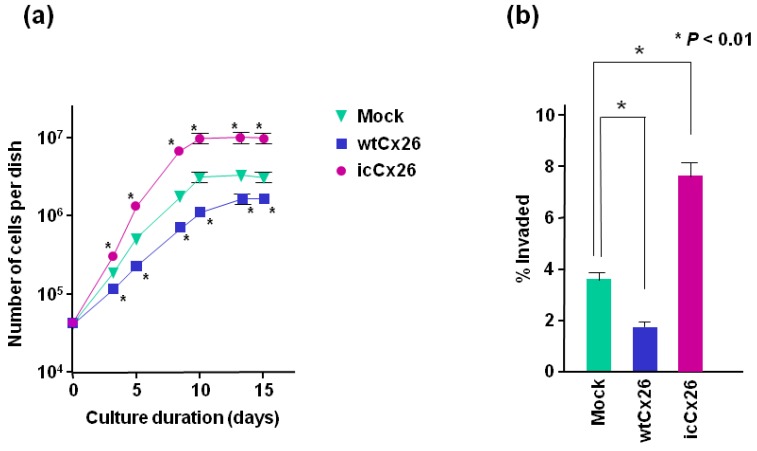
Effects of wtCx26 and icCx26 proteins on cell proliferation and invasion capacity in vitro of FaDu cells. (**a**) Growth curve of each clone of FaDu cells. The wtCx26, icCx26, and mock clones were cultured for the indicated periods. The cells were counted every 2 or 3 days in triplicate dishes. Error bars represent the SD (*n* = 3). No error bar is indicated when the SD is too small to show. * *p* < 0.001 (significantly different from the mock clone at the corresponding time point). (**b**) Invasion capacity of each clone into the matrix basement membrane. The cells were seeded onto Matrigel, which had been settled on cell culture inserts in advance. The cells that infiltrated into the Matrigel layer were counted and their proportion to the total cell number is indicated. Error bars represent the SD (*n* = 6).

To examine the effects of intracellular accumulation of Cx26 protein on cell proliferation, each clone transduced with wtCx26, icCx26, or the mock construct was plated in 60-mm dishes in triplicate and the cell number was counted with hemocytometer. As shown by growth curve (Figure 3a), the proliferation rate of FaDu cell clone overexpressing wtCx26 protein is significantly lower than that of the mock clone. More interestingly, overexpression of icCx26 protein retained in a Golgi area has remarkably elevated the proliferation rate compared with the mock clone. It has been known that GJ-mediated modulation of cell proliferation is often most obvious in the alteration of saturation density [23,24]. It is also the case with our experiments, i.e., the saturation densities of wtCx26- and icCx26-transduced clones are approximately 60% and 180% of that of the mock clone, respectively (Figure 3a). Taken together, while cell proliferation is suppressed by Cx26 protein integrated into GJ, GJ-independent Cx26 protein localised in a Golgi area enhances cell proliferation.

We further investigated whether overexpression of icCx26 protein could affect invasiveness of FaDu cells by evaluating the ability of each clone to invade the basement membrane matrix. Similarly to other malignant phenotypes, Figure 3b demonstrates that overexpression of icCx26 and wtCx26 proteins enhances and declines the invasiveness of FaDu cells, respectively.

### 2.4. icCx26 Protein Reinforces Tumorigenicity of FaDu Cells in Nude Mice

**Figure 4:**
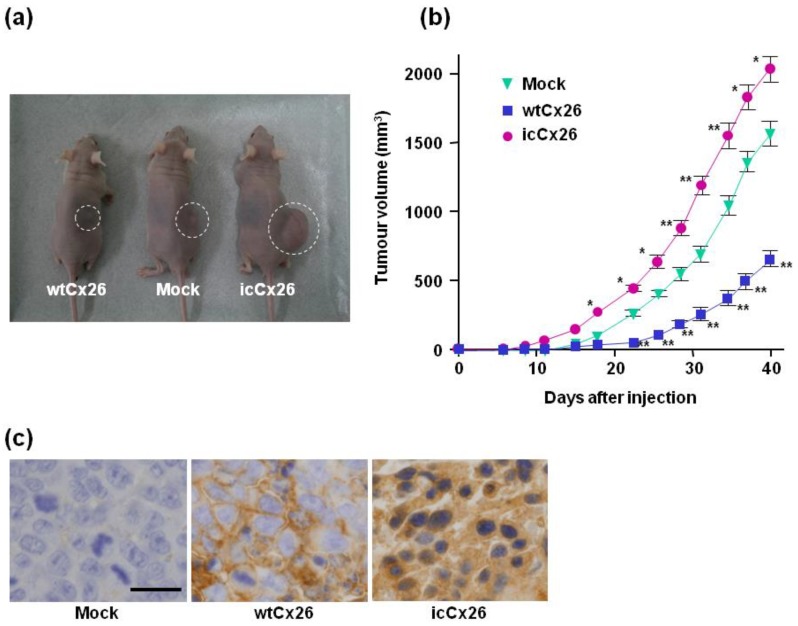
Xenografts of the wtCx26, icCx26, and mock clones into nude mice and tumorigenicity assay in vivo. (**a**) Representative mice bearing tumours raised from 1 × 10^6^ cells of each clone. (**b**) Tumorigenicity in vivo of each clone. The size of each tumour was measured every 2 or 3 days. Error bars represent the SD (*n* = 6). No error bar is indicated when the SD is too small to show. * *p* < 0.03, ** *p* < 0.001 (significantly different from the mock clone at the corresponding time point). (**c**) Expression and subcellular localisation of Cx26 protein in the tumours raised from the xenografts. As revealed by immnohistochemistry, wtCx26 was localised in a cell-cell boundary area. Scale bar, 20 µm.

To assess the effect of intracellular accumulation of Cx26 protein on tumorigenicity in vivo, 1 × 10^6^ cells each of the three clones transduced with wtCx26, icCx26, or the mock construct were implanted subcutaneously into the backs of 6 male nude mice per clone. All of the 18 mice examined developed xenograft-derived subcutaneous tumours (Figure 4a). The growth curves of tumours show that the clone overexpressing icCx26 protein manifests a greatly higher growth rate of tumours compared with the mock clone (Figure 4a,b). Consistent with many other papers, the growth rate of tumours was significantly declined by overexpression of wtCx26 protein (Figure 4b), which forms GJ plaques at a cell-cell contact area (Figure 1b).

Furthermore, the tumours derived from each clone were subjected to immunohistochemistry to determine subcellular localisation of Cx26 protein in the tumours. Figure 4c shows that wtCx26 and icCx26 proteins are localised in cell membrane and cytoplasm, respectively, in the corresponding tumours. Regardless of expressed types of Cx26 protein, the behaviours of the Cx26 proteins examined are not different between in vitro and in vivo. These results clearly indicate that oncogenic roles of icCx26 protein have been confirmed both in vitro and in vivo.

## 3. Discussion

It has been established by many convincing evidences that GJ is, in general, a tumour-suppressive cellular apparatus. As such, are connexin proteins, an exclusive component of GJ, considered to be a tumour suppressor? When connexin proteins serve as GJ components, they are usually tumour-suppressive. However, connexin proteins often translocate from cell membrane into an intracellular site in different histological types of malignant tumours [14]. Although such an aberrantly-localised connexin protein cannot function as GJs, their intracellular translocation might generate an unexpected intrinsic function in connexin molecules and make some contribution to tumour progression. To address such a question, we have previously demonstrated that Cx32 protein is not localised in plasma membrane but in the Golgi-apparatus in human HuH7 hepatoma cells and that accumulation of Cx32 protein in the Golgi-apparatus reinforces different malignant phenotypes of HuH7 cells, resulting in the induction of metastasis in the mice xenografted with HuH7 cells overexpressing Cx32 protein in the Golgi-apparatus [17,18].

HuH7 cells are, by nature, incapable of forming GJs due to retention of Cx32 protein in the Golgi-apparatus. In the present study, we employed FaDu HNSCC cells, which express almost no connexin protein, but which can support membrane sorting of a normal connexin protein to generate functional GJs (Figure 1). In other words, FaDu cells are quite normal in terms of the GJ system. HNSCCs are raised from the basal cells, which express mainly Cx26 among different connexin proteins, in the stratified squamous epithelium. Therefore, using FaDu cells and the mutant Cx26 construct coding Cx26 protein conjugated with an ER-Golgi retention signal, we could successfully compare functions between icCx26 and wtCx26 proteins in terms of GJIC, cell proliferation, invasion, and tumorigenicity and find out that icCx26 protein had a GJ-independent intrinsic oncogenic function.

We have been unable to provide the mechanism of how our icCx26 protein behaves in a pro-oncogenic manner. Since icCx26 protein is localised in a Golgi area, ER-stress response may be involved in the mechanism. ER-stress induces two contradictory responses called “adaptive response” and “destructive response” [25]. The adaptive response can be pro-oncogenic. While many proteins related to ER-stress response function in a Golgi-independent manner, ER-resident ATF6 protein is translocated to Golgi apparatus, activated there, then imported into nucleus, and finally induces ER-stress response [26]. icCx26 protein might be involved in such a pathway. More directly, icCx26 protein might play a role in recently-unravelled Golgi stress [27].

It has long been proposed that connexin in tumour has dual or even multiple functions [28,29]. Although intracellular connexin proteins including intra-Golgi, cytoplasmic, and nuclear connexins are rather common in tumours [30,31,32,33], there has been little examination thus far of their existence and roles in a physiological condition [34]. Thus, this study has contributed to proving a pathological significance of intracellular connexin proteins. Furthermore, membrane-sorting mechanism of connexin proteins still remains controversial. From aspects of cancer control, mechanism of intrinsic function of connexin proteins in a pathological condition and improvement of membrane sorting of connexin proteins should become targets to be elucidated.

## 4. Materials and Methods

### 4.1. Vector Construct

To add the amino acid sequence AKKFF, an ER-Golgi retention signal [19], to C-terminus of Cx26, the fragment containing the coding sequence of human Cx26 (*GJB2*) cDNA [35] was amplified by polymerase chain reaction with the following set of primers: Forward, 5′-ACACAAGCATCTTCTTC-3′; Reverse, 5′-GCGAATTCTTAGAAGAACTTCTTGGCAACTGGCTTTTTTGACTTCCCAGA-3′. It was then digested by the restriction enzymes Bsp119I and EcoRI. The Bsp119I-EcoRI fragment was exchanged with the corresponding fragment of the previously prepared human Cx26/pQCXIN construct, resulting in the mutant Cx26 cDNA, coding icCx26 protein, cloned into pQCXIN retrovirus vector (Clontech Laboratories, Mountain View, CA, USA).

### 4.2. Cell Culture and Retroviral Transduction

**Figure A2:**
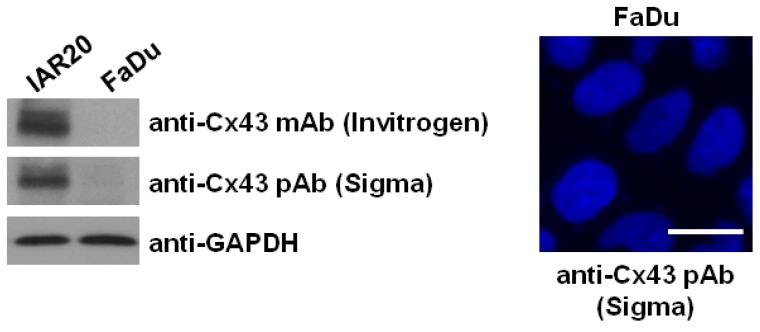
Expression of Cx43 protein in FaDu cells. Immunoblotting was performed with two different anti-Cx43 antibodies, mAb (Invitrogen; clone CX-1B1) and pAb (Sigma-Aldrich). As a positive control, IAR20 cells were used. For indirect immunofluorescence, the fluorescent signals of Cx26 protein were visualised by Alexa-488. Nuclei were stained with DAPI. Note that no positive signals for Cx26 protein are detected. Scale bar, 20 µm.

**Figure A3:**
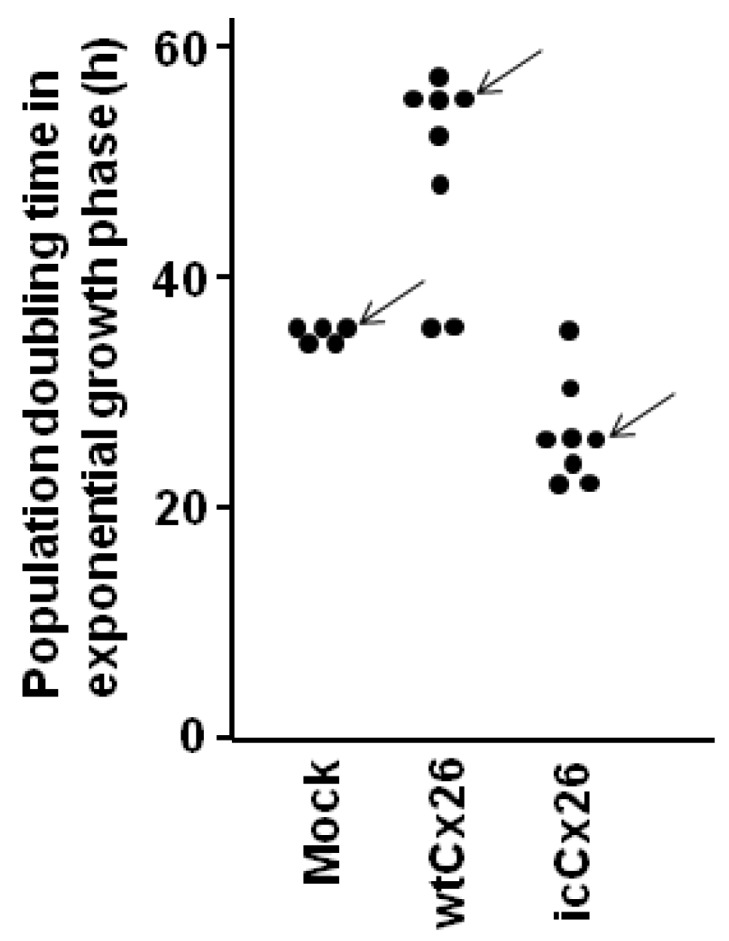
Population doubling time of randomly-selected clones of stable transductants. Randomly-selected 8 clones each from wtCx26 and icCx26 stable transductants as well as 5 clones from mock transductants were subjected to a preliminary cell proliferation assay to measure population doubling time. Each plot corresponds to a single clone. Note that none of the mock clones show native clonal variation. 2 and 1 clones of wtCx26 and icCx26 transductants, respectively, show a population doubling time indistinguishable from that of the mock clones. It has been confirmed that these 3 clones express Cx26 protein at a similar level to that of parental FaDu cells as revealed by immunoblotting. Therefore, omitting the 3 clones, a clone (arrow) indicating a median value was chosen from each of three groups for later experiments. Clonal variation between the clones is due to difference in expression levels of the transgene products.

Human FaDu hypopharyngeal squamous cell carcinoma cell line was supplied by American Type Culture Collection (ATCC, Manassas, VA, USA). It has been confirmed that our FaDu cells express no detectable level of Cx43 protein as revealed by immunoblotting and immunofluorescence (Figure A2). The cells and their established subclones were cultured in RPMI1640 medium (Nissui Pharmaceutical, Tokyo, Japan) containing 10% foetal calf serum (FCS), 100 units/mL penicillin and 100 µg/mL streptomycin. PT-67 packaging cells were grown in Dulbecco modified Eagle medium (Thermo Fisher Scientific, Rockford, IL, USA), 10% FCS, 100 U/mL penicillin and 100 µg/mL streptomycin. All the cells were incubated at 37 °C in a humidified atmosphere containing 5% CO_2_ in air. To determine cell proliferation, 5 × 10^4^ cells were seeded into 60-mm dishes in triplicate in 4 mL of medium with 10% FCS. The cells were grown under the aforementioned conditions and counted every 2 or 3 days with a haemocytometer. Dead cells, as determined by trypan blue staining, were left out of the count. FaDu cells expressing wtCx26 or icCx26 protein were established as follows. wtCx26/pQCXIN, icCx26/pQCXIN construct, or pQCXIN empty vector was transfected with FuGENE HD Transfection Reagent (Promega, Madison, WI, USA) into the packaging cell PT-67, and stable transformants were selected with 400 µg/mL G418. FaDu cells were then infected with virus-containing supernatant, supplemented with 4 µg/mL of polybrene, from PT-67 cells transfected with each of the 3 constructs. After 3 weeks of selection with 400 µg/mL G418, G418-resistant FaDu transductants were subcloned by limiting the dilution method. Randomly-selected 8 clones each from wtCx26 and icCx26 stable transductants as well as 5 clones from mock transductants were subjected to a preliminary cell proliferation assay to measure population doubling time. Since all the mock clones showed similar population doubling times without variation, native clonal variation of FaDu cells is considered to be small (Figure A3). 2 and 1 clones of wtCx26 and icCx26 transductants, respectively, showed a population doubling time indistinguishable from that of the mock clones (Figure A3). Omitting these 3 clones, we used a clone indicating a median value from each of three groups for later experiments. It has been confirmed by immunoblotting that the omitted 3 clones express no exogenous Cx26 protein.

### 4.3. Immunoblotting

Immunoblotting analysis was performed mostly as previously described [23]. As primary antibodies, anti-Cx26 polyclonal antibody (pAb) (Thermo Fisher Scientific) and anti-GAPDH monoclonal antibody (mAb) clone 6C5 (HyTest, Turku, Finland) were applied after diluted at 1:500 and 1:10,000, respectively. Then as second antibodies, horseradish peroxidase (HRP)-conjugated anti-rabbit and anti-mouse IgG antibodies (GE Healthcare Bio-Sciences, Piscataway, NJ, USA) were applied at dilution ratios 1:2000 and 1:5000, respectively. Finally, the protein-antibody complex was chemiluminated with a WEST-one Western Blot Detection System (iNtRON Biotechnology, Seoul, Korea) following the manufacturer’s protocol.

### 4.4. Indirect Immunofluorescence

Indirect immunofluorescence was performed as described previously. Anti-Cx26 mAb clone CX-12H10 (Thermo Fisher Scientific) and anti-p120 Catenin pAb (Sigma-Aldrich, St. Louis, MO, USA), anti-GM130 pAb (Sigma-Aldrich) were diluted at 1:150, 1:200, and 1:3500, respectively. After fixation with acetone, cells are incubated with the diluted primary antibodies. Specific signals were revealed by anti-mouse IgG-Alexa 488 (Thermo Fisher Scientific) and anti-rabbit IgG-Alexa 568 (Thermo Fisher Scientific). Nuclei were stained with DAPI (KPL, Gaithersburg, MD, USA) at a concentration of 0.5 µg/mL.

### 4.5. Scrape-Loading Dye-Transfer Assay

The assay was performed as described in el-Fouly et al. [36] with modification. The confluent cells on 60-mm dishes were washed with PBS containing 1 mM CaCl_2_ and immersed in 3 mL of dye cocktail composed of 0.1% Lucifer yellow CH (Sigma-Aldrich) and 0.1% RITC-dextran (Sigma-Aldrich). Several parallel scrape lines were then made with a micropipette tip, and the cells were incubated for 5 min at 37 °C. After rinsed with PBS, the cells dye-coupled with Lucifer yellow were detected under a fluorescence microscope. The cells positive for RITC-dextran were considered to be primarily-scraped but not dye-coupled cells.

### 4.6. Invasion Assay

Invasion capacity was evaluated quantitatively with Falcon Permeable Support with 8-μm-pore filter (Corning Inc. Life Sciences, Tewksbury, MA, USA). The filters of cell culture inserts were precoated with 500 μg Matrigel (Corning Inc. Life Sciences), dried for 24 h in an incubator and rehydrated by RPMI1640 for 1 h before inoculation of cells. The cell culture inserts were set on 4 mL of FCS-supplemented RPMI1640 poured for the lower compartments of 6-well plates. 5.0 × 10^5^ cells resuspended in 2 mL of serum-free RPMI1640 containing 0.01% bovine serum albumin were seeded to each upper well. After 72 h of incubation, cells were trypsinised and collected separately from the top of the membrane, the underside of the membrane, and the lower compartment. Invasion was quantified as the percentage of cells recovered from the underside of the membrane and the lower compartment over the total cell number.

### 4.7. Xenograft into Nude Mice

1 × 10^6^ cells suspended in 200 μL of PBS were injected subcutaneously into the backs of 6 male BALB/c-nu/nu mice of 6 weeks of age per clone. Two perpendicular diameters (*d*_1_ and *d*_2_) of each tumour were measured every 2 or 3 days and converted to tumour volume (mm^3^) according to the formula: V = (π/6)(*d*_1_ × *d*_2_)^3/2^ [37]. 40 days after injection, the mice were euthanized. A portion of each tumour was frozen, and the rest was fixed in 10% buffered formalin for further analysis. The protocol of the animal work was approved (No. 14016, 31/Jan/2014) by the Committee for Ethics of Animal Experimentation and in accordance with the Guidelines of Animal Experiments of Akita University.

### 4.8. Immunohistochemistry

Formalin fixed paraffin-embedded sections on slide glass were deparaffinised, then immersed in 3% hydrogen peroxide/methanol at room temperature for 15 min. The slides were incubated with anti-Cx26 mAb clone CX-12H10 (Thermo Fisher Scientific) in a humidified chamber at 4 °C overnight. Specific signals were visualised by employing HRP-labelled polymer method as follows, the slides were reacted with EnVision+ system-HRP for mouse (Agilent, Santa Clara, CA, USA) at room temperature for 30 min and finally 3,3′-diaminobenzidine was oxidized for signal detection.

### 4.9. Statistical Analysis

The student’s *t*-test was performed for the estimation of statistical significance. *p* values are two-tailed. All experiments were independently repeated at least 3 times except for the tumorigenicity assay of xenografts in mice, which was performed only once.

## Abbreviations

GJ	gap junction
GJIC	gap junctional intercellular communication
HNSCC	head and neck squamous cell carcinoma
wtCx26	wild-type connexin26
icCx26	intracellular conexin26
ERGIC	ER-Golgi intermediate compartment
PBS	phosphate-buffered saline
GAPDH	glyceraldehyde-3-phosphate dehydrogenase
HRP	horseradish peroxidase
DAPI	diamidine phenylindole dihydrochloride
SD	standard deviation
